# Language and communication non-pharmacological interventions in patients with Alzheimer's disease: a systematic review. Communication intervention in Alzheimer

**DOI:** 10.1590/1980-57642016dn11-030004

**Published:** 2017

**Authors:** Aline Nunes da Cruz Morello, Tatiane Machado Lima, Lenisa Brandão

**Affiliations:** 1 Instituto de Psicologia, Universidade Federal do Rio Grande do Sul – UFRGS – Porto Alegre, RS, Brazil.; 2 Departamento de Saúde e Comunicação Humana, Universidade Federal do Rio Grande do Sul – UFRGS – Porto Alegre, RS Brazil.

**Keywords:** communication, language, rehabilitation, alzheimer's disease, review, comunicação, linguagem, reabilitação, doença de alzheimer, revisão

## Abstract

Alzheimer's disease considerably compromises communication skills. Language changes become more prominent as the disease progresses. Deterioration of language and cognition reduces the ability of holding conversations, which has a negative impact on social interaction.

**OBJECTIVE:**

To conduct a systematic review of the literature for articles reporting interventions focused on the language and communication of people with Alzheimer's disease (AD) without use of medication.

**METHODS:**

We performed a search using the keywords Alzheimer's disease, language, communication, cognition, cognitive intervention, rehabilitation and therapy, and their corresponding Portuguese and Spanish terms, on the SciELO, LILACS, PubMed and PsychINFO databases. We analyzed intervention studies published from 1993 to 2016 that involved stimulation of language skills and/or communication with pre-and post-intervention quantitative results, and whose samples included at least 50% with a diagnosis of probable AD. Studies were analyzed and classified into four levels of evidence, according to the criteria described in the literature.

**RESULTS:**

Twenty-eight articles were included. The majority of the designs had medium-to-low scientific evidence. Most interventions showed benefits for at least one language or communicative skill. Eight types of interventions emerged from the analysis of the studies. Further research with higher levels of evidence is recommended in the investigation of interventions focused on language and communication skills of patients with dementia.

**CONCLUSION:**

Studies with high levels of evidence on the topic investigated are only being conducted on a small scale. Two intervention techniques seem potentially effective: lexical-semantic approaches and interventions that work with different cognitive skills (including language).

## RESUMO

A doença de Alzheimer (DA) compromete consideravelmente as habilidades de comunicação. As mudanças de idioma se tornam mais proeminentes à medida que a doença avança. A deterioração da linguagem e da cognição reduz a capacidade de manter conversas, o que tem um impacto negativo na interação social.

**OBJETIVO:**

Realizar uma revisão sistemática da literatura para artigos que denunciam intervenções focadas na linguagem e comunicação de pessoas com DA sem uso de medicação.

**MÉTODOS:**

Realizamos uma pesquisa usando as palavras-chave doença de Alzheimer, linguagem, comunicação, cognição, intervenção cognitiva, reabilitação e terapia, e seus correspondentes termos em português e espanhol, nas bases de dados SciELO, LILACS, PubMed e PsychINFO. Analisamos os estudos de intervenção publicados de 1993 a 2016 que envolveram estimulação de habilidades linguísticas e / ou comunicação com resultados quantitativos pré e pós-intervenção e cujas amostras incluíram pelo menos 50% com diagnóstico de DA provável. Os estudos foram analisados ​​e classificados em quatro níveis de evidência, de acordo com os critérios descritos na literatura.

**RESULTADOS:**

Vinte e oito artigos foram incluídos. A maioria dos projetos tinha evidências científicas de médio a baixo. A maioria das intervenções mostrou benefícios para pelo menos uma linguagem ou habilidade comunicativa. Oito tipos de intervenções emergiram da análise dos estudos. Pesquisas adicionais com maiores níveis de evidência são recomendadas na investigação de intervenções focadas em linguagem e habilidades de comunicação de pacientes com demência.

**CONCLUSÃO:**

Estudos com altos níveis de evidência sobre o tema investigado estão sendo conduzidos apenas em pequena escala. Duas técnicas de intervenção parecem ser efetivas: abordagens lexical-semânticas e intervenções que funcionam com diferentes habilidades cognitivas (incluindo linguagem).

## INTRODUCTION

Alzheimer's disease considerably compromises communication skills. Language changes become more prominent as the disease progresses. During initial stages, deficits may manifest through lexical access difficulties, with mild changes on the phonological and grammatical level.^1^ This stage is marked by the presence of anomies and replacement of words, such as the use of general terms and the presence of semantic paraphasia.^1^^,^ 2 Sentences produced may have reduced complexity and discrete grammatical errors.^3^^-^ 5 During the moderate stage, discourse becomes increasingly affected with circumlocutions and repetitions.^6^ Coherence is highly affected, with reduced conversational turns,^7^ sudden topic shifts^6^ and, in moderately severe stages, a lack of awareness of mistakes in conversations is observed.^8^ At advanced stages, AD patients may reach complete mutism.^1^ Communication deficits are strongly related both with decline in the semantic system^1^^,^ 6 and with extra-linguistic cognitive deficits.^9^ Deterioration of language and cognition reduces the ability of holding conversations, which has a negative impact on social interaction.^10^

Pharmacological treatment does not arrest brain deterioration, although currently available treatments seek to promote some cognitive maintenance and reduction in behavioral symptoms.^11^ The main purpose of communication intervention is to optimize the adaptation of cognitive, communicative and behavioral functioning to the environment. However, a growing number of studies are investigating the possibilities of promoting the maintenance and improvement of cognitive abilities.^12^ Neuropsychological interventions promote the use of compensatory strategies, training of cognitive skills and adaptation to permanent losses.^13^

Although there is some scientific evidence that language and communication interventions may offer benefits for people with AD, there is clearly a lack of investigations on the theme. Only a few reviews specifically discuss language and communication interventions for the population with AD.^10^^,^ 14 In addition, there is a gap in the literature in relation to surveying the scientific criteria adopted in these investigations. Further research in this area is needed so that the best evidence available on research can be integrated into professional practice, offering better quality of services.^15^

The general objective of this study was to conduct a systematic review on therapeutic non-pharmacological interventions that aim to maintain or rehabilitate the language and communicative skills of AD patients. Articles were classified according to their scientific evidence levels and to the intervention methods used. Outcomes were described and the presence of post-intervention follow-ups was considered.

## METHODS

The search in the literature was conducted using the following keywords in English, Portuguese and Spanish, respectively: *Alzheimer's disease, language, communication, cognition, cognitive, intervention, rehabilitation* and *therapy. Doença de Alzheimer, linguagem, comunicação, cognição, cognitiva, intervenção, reabilitação* and *terapia. Enfermedad de Alzheimer, lenguaje, comunicación, cognición, intervención, rehabilitación* and *terapia*. Different combinations of these terms were used during the search. The bibliographic research was conducted on the following databases: Pubmed, PsychINFO, Scielo, and LILACS. Studies published between 1993 and 2016 that were electronically available on the databases were analyzed.

The search for articles was conducted by two reviewers, together and independently, and consisted of the following stages: [1] Selection based on the title of the manuscript: electronic search on the databases of papers with titles that seemed to be related to the systematic review; [2] Selection based on the abstract of the manuscript: the articles selected by title were screened from the analysis of the abstracts. In order for the study to be included in the article sample, it had to include the following information: at least 50% of the sample with an AD diagnosis; study the effect of a non-pharmacologic intervention for patients with dementia, involve an intervention that included the stimulation of language and/or communication skills; the study had to include quantitative results; and report pre- and post-intervention results; [3] After verifying whether the inclusion criteria were actually met, the manuscript was analyzed in full. This screening consisted of retaining in the sample those articles that met the criteria while excluding studies published in languages other than Portuguese, English or Spanish, as well as literature reviews, meta-analysis studies and duplicate publications. After the selection, the reviewers met to debate on the inclusion or exclusion of articles that raised doubts during the independent selection process. The disagreements were resolved by discussion and consensus.

The articles selected were analyzed and classified according to scientific evidence levels. The interpretation was based on the adaptation of criteria described in the neuropsychological literature.^16^ Four evidence levels were adopted. Level I included prospective design studies, randomized studies, controlled clinical trials, blind evaluation of results in a representative population with: pre- and post-intervention measurement of at least one clearly defined communicative and/or linguistic function of the samples, inclusion/exclusion criteria in the experimental and control samples, cognitive pairing of the experimental and control groups during the pre-intervention phase, minimal number of participants with an adequate drop out estimation, report on the relevant characteristics at baseline, matching of comparative groups for variables such as age and education. Level I may have even more advanced characteristics in relation to the control of variables, such as random allocation of participants into the experimental and control samples and confidentiality in the allocation. Level II included cohort studies and randomized clinical trials of a representative sample of the population. Studies classified as level II did not meet any of the level I criteria. Level III studies constituted all other controlled trials (including representative sample of the population, the use of controls and patients that were used as their own controls), whose results were independently evaluated or derived from objective measurements, reducing the possibility of being affected by biases. Level IV consisted of multiple and case studies, and uncontrolled investigations whose evidence was based on the opinion of specialists. Other important factors analyzed included the quality and detail of description of intervention methods.

## RESULTS

Titles from 7,914 articles were identified on the electronic searches. Of these, 165 articles were identified as potentially appropriate for review and the respective abstracts were read. Only 29 were related to our investigation and met the inclusion criterion, and these were read in full. Among the excluded articles, the most common reasons for exclusion were: duplicate publications, no results shown on language and/or communication skills, non-inclusion of pre- and post-intervention results specifically for these skills, lack of specification of the types of dementia included on the sample, in addition to, in some cases, not constituting an intervention study.

Different types of intervention were observed from the articles included in this review. Among these, nine articles focused mainly on the intervention of different cognitive skills (including language), six on language activities integrated into physical activity, six on a lexical-semantic approach, three on face-name association interventions, two on the use of "memory cards" during conversation, one on an instrumental communicative activity of daily living (talking on the phone) through the use of mnemonic assistance, one conversational intervention, and one on the training of the communicative skills of caregivers of people with AD (Chart 1). Of these, only one study investigated the effects of a therapeutic intervention associated with pharmacological treatment in comparison to treatment with a pharmacological intervention alone.^17^ All the other articles only mentioned whether participants were using medication or not.

**Chart 1:** Summary of papers included in the sample by author, level of evidence, description of the intervention and results.

Study	EL	Description of the intervention	Results
**Intervention on different cognitive skills (including language)**			
Quayhagen, Quayhagen, Corbeil, Hendrix, Jackson, Snyder et al. (2000)29	I	• n (AD)= non-specific AD stage (mild to moderate). • Technique and task used: four types of intervention (cognitive stimulation, didactic counseling, support seminar group for dyads and Initial Stage Care Program). • Treatment method: individual and dyad cognitive stimulation. • Frequency and duration of the sessions: five times a week, one hour each session (cognitive stimulation). • Treatment period: eight weeks. • Work with caregivers involved: yes. • Use of associated medication: no.	Significant improvements in post-intervention results for delayed memory and verbal fluency. Improved caregiver-patient interaction. There was a decline in the control group.
Davis, Massman & Doddy (2001)26	II	• n (AD)= 37. AD stage: not specified in the study • Technique and task used: face-name association combined with spaced retrieval and attention exercises. • Treatment method: individual. • Frequency and duration of the sessions: one-hour weekly sessions. • Treatment period: five weeks. • Work with caregivers involved: no. • Use of associated medication: yes.	Significant increase for face-name association. Verbal fluency improvement observed (animals).
Cipriani, Bianchetti & Trabucchi (2006)30	III	• n (AD)= 10. AD stage: not mentioned. • Technique and task used: computer software with exercises to stimulate different cognitive skills. • Treatment method: individual. • Frequency and duration of the sessions: four times a week, with 13 to 45-minute sessions. • Treatment period: four weeks. • Work with caregivers involved: no. • Use of associated medication: yes.	Verbal fluency improvements. Significant increase in verbal production, semantic categorization, verbal understanding and phonological discrimination.
Tsantali, Economidis (2014)25	IV	• n (AD)= 1. AD stage: mild. • Technique and task used: errorless learning. Several language activities, such as semantic categorization and naming. • Treatment method: individual. • Frequency and duration of the sessions: 1^st^phase: four months, five times a week, three of them with direct supervision by the therapist, 90 minutes. 2^nd^phase: one year, three times a week with discrete supervision by the therapist, 90 minutes. 3^rd^, 4^th^ and 5^th^phases: minimal involvement of the therapist. • Treatment period: 5 years. • Work with caregivers involved: no. • Use of associated medication: no.	Significant improvement in verbal fluency, writing, written understanding. Follow-up indicated the maintenance of the language improvements obtained.
Ávila (2003)11	IV	• n (AD)= 1. AD stage: mild. • Technique and task used: errorless learning. • Treatment method: individual and in groups. • Frequency and duration of the sessions: weekly sessions. 90 minutes for group sessions and one hour for individual sessions. • Treatment period: 22 months. • Work with caregivers involved: yes. • Use of associated medication: yes.	The reading and writing (after individual therapy) returned to the normality range. Gradual improvements in naming throughout the years. Semantic verbal fluency and animals were stable.
Abrisqueta-Gomez, Canali, Vieira, Aguiar, Ponce, Brucki et al (2004)20	IV	• n (AD)= 3. AD stage: mild to moderate. • Technique and task used: errorless learning, clue vanishing. • Treatment method: individual and in groups. • Frequency and duration of the sessions: twice a week (one for each treatment method). 90 minutes for group sessions and one hour for individual sessions. • Treatment period: 24 months. • Work with caregivers involved: no. • Use of associated medication: yes.	Improvements in phonemic fluency, understanding, and repetition but decline in semantic fluency. During the second year after the intervention, decline was verified and, in some cases, these skills were maintained. The improvements obtained did not persist through the second year, showing the progressive nature of the disease.
Oullell, Bruna & Puyuelo (2006)31	IV	• n (AD)= 1. AD stage: mild. • Technique and task used: psycho stimulation and phonoaudiological intervention. • Treatment method: group psycho stimulation and individual phonoaudiological stimulation. • Frequency and duration of the sessions: not mentioned. • Treatment period: 24 months. • Work with caregivers involved: yes. • Use of associated medication: yes.	Articulation, fluency, understanding, automatic speech and naming were stable. After two years, a decline occurred. Reading and writing were preserved over time.
Bottino, Carvalho, Alvarez, Avila, Zukauskas, Bustamante et al. (2002)19	IV	• n (AD)= 6. AD stage: mild. • Technique and task used: mnemonic assistance and errorless learning. • Treatment method: individual, in groups and at home. • Frequency and duration of the sessions: weekly sessions. Individual and at home: 40 minutes. Group sessions: one hour. • Treatment period: 22 weeks. • Work with caregivers involved: yes. • Use of associated medication: yes.	Statistically significant results were not obtained. Although not significant, there was a tendency toward improvement for cognitive and functional deficits. Maintenance of the language and writing scores was observed, as well as a reduction in the understanding scores and a slight increase for writing and naming.
Ramström (2011)32	IV	• n (AD)= 5. AD stage: mild to moderate. • Technique and task used: exercises to stimulate different skills. Computer used. • Treatment method: individual. • Frequency and duration of the sessions: four to five hours, twice a week. • Treatment period: 12 months. • Work with caregivers involved: no. • Use of associated medication: not mentioned.	Maximum scores for reading understanding and reading out loud tasks, and these results were maintained for 12 months. Non-generalized improvement and maintenance for listening comprehension, naming, syntax, repetition and writing.
**Language intervention integrated into physical activities**			
Cott, Dawson, Sidani& Well (2002)33	I	• n (AD)= 3. AD stage: not mentioned in the study • Technique and task used: three programs (walking and talking, talking only, and no intervention). • Treatment method: individual. • Frequency and duration of the sessions: five weekly session of 30 minutes each. • Treatment period: 16 weeks. • Work with caregivers involved: yes. • Use of associated medication: not mentioned.	Significant improvement in social and global communication for the conversation group. Maintenance of social communication for the walking-and-talking group, and no effects shown for global communication.
Tappen, Williams, Barry & DiSesa (2002)34	I	• n (AD)= 15. AD stage: not mentioned. • Technique and task used: three programs (conversation, assisted walking and conversation and walking). • Treatment method: individual. • Frequency and duration of the sessions: three weekly sessions of 30 minutes each. • Treatment period: 16 weeks. • Work with caregivers involved: no. • Use of associated medication: not mentioned.	All groups showed a decline in discourse skills (number of words produced, informative units and concision). Only some participants showed a non-significant improvement; for these individuals, it was observed that the scores obtained in the "conversation" group indicated better results.
Arkin (2007)35	III	• n (AD)= 24. AD stage: mild to moderate. • Technique and task used: physical, language, and memory exercises, and voluntary activities. • Treatment method: individual. • Frequency and duration of the sessions: two sessions a week. • Treatment period: maximum of four years. • Work with caregivers involved: no. • Use of associated medication: not mentioned.	Improvement or maintenance of speech. Lower declines in verbal fluency and naming measurements over time. Higher maintenance of skills in individuals that completed two or more years of intervention.
Mahendra & Arkin (2003)36	III	n (AD = 4. AD stage: mild to moderate. • Technique and task used: physical, linguistic-cognitive and community activities. • Treatment method: not mentioned. • Frequency and duration of the sessions: two to two and a half hours. Community activities of one to two hours • Treatment period: four years. • Work with caregivers involved: no. • Use of associated medication: no.	Improvement and maintenance in discourse skills, however, this was not generalized to all participants.
Arkin (2001)37	III	• n (AD)= 11. AD stage: mild to moderate. • Technique and task used: physical exercises, language and memory stimulation. • Treatment method: not mentioned. • Frequency and duration of the sessions: twice a week • Treatment period: ten weeks. • Work with caregivers involved: no. • Use of associated medication: not mentioned.	Equivalent performances in the experimental and control groups. Significant results were not obtained on language and discourse skill tests.
Rue, Felten & Turkstra (2016)38	III	• n (AD)= 42. AD stage: mild AD. • Technique and task used: physical exercises and language stimulation. • Treatment method: sessions with a trained volunteer and an individual with dementia, with exercise and language stimulation sessions interspersed with social or volunteer outings • Frequency and duration of the sessions: twice a week, one and a half-hour session • Treatment period: first follow-up 10.65 months and second follow-up at an average of 20.55 months • Work with caregivers involved: no. • Use of associated medication: not mentioned.	Remained stable in cognitive function, mood, and physical fitness through an initial follow-up. A small subgroup that completed a second follow-up at an average of 20 months continued to perform near baseline levels.
**Lexical-semantic therapy**			
Jelcic et al. (2014)40	I	• n (AD)= 27. AD stage: mild. • Technique and task used: activities focused on interpreting written words, sentences and stories. One group conducted these activities face to face and the other by teleconference. • Treatment method: Groups: 1) Lexical-semantic stimulation with teleconference technology. 2) Face to face lexical-semantic stimulation. 3) Non-structured cognitive stimulation. • Frequency and duration of the sessions: twice a week, one-hour sessions. • Treatment period: three months. • Work with caregivers involved: no. • Use of associated medication: no.	Improvement in language scores that measure phonemic and verbal fluency for the group that underwent stimulation with the use of teleconferencing. Follow-up not conducted.
Jelcic, Cagnin, Meneghello, Turolla, Ermani & Dam (2012)39	I	• n (AD)= 40 AD stage: mild. • Technique and task used: interpretation of isolated words, in sentences and stories. • Treatment method: small groups with four participants • Frequency and duration of the sessions: twice a week, one-hour sessions. • Treatment period: three months. • Work with caregivers involved: no. • Use of associated medication: no.	Significant improvement in language scores (naming). No improvement in the control group. Phonemic and semantic fluency improvement (control and experimental group). The follow-up indicated decline, however, performance was still superior to baseline.
Ousset, Viallard, Puel, Celsis, Démonet, Cardebat (2002)41	II	• n (AD)= 16. AD stage: not mentioned in the study • Technique and task used: narratives shown on a computer. Patients were told to produce the words which corresponded with the definition visualized on the screen. • Treatment method: not mentioned. • Frequency and duration of the sessions: weekly 45-minute sessions. • Treatment period: 16 weeks. • Work with caregivers involved: no. • Use of associated medication: yes.	Significant improvement on naming and post-intervention scores and reduction on the control group.
Rothi et al. (2009)21	III	• n (AD)= 6. AD stage: not mentioned. • Technique and task used: errorless learning. Visual stimulation associated with word. • Treatment method: individual. • Frequency and duration of the sessions: 60 minutes, four days a week. • Treatment period: 20-35 sessions. • Work with caregivers involved: no. • Use of associated medication: yes.	Significant improvements in post-intervention naming for three participants (total of six); of these individuals, two had a significant improvement on the generalization testing.
Noonan, Pryer, Jones, Burns e Ralph (2012)22	III	• n (AD)= 8. AD stage: not mentioned. • Technique and task used: errorless learning and trial and error. Images associated with word. Treatment method; individual. • Frequency and duration of the sessions: twice a week, 40-60 minutes each session. • Treatment period: five weeks. • Work with caregivers involved: no. • Use of associated medication: no.	Significant improvements in naming (errorless learning and trial and error group) and superior results to the subjects in the control group (no treatment). The follow-up indicated superior naming results relative to baseline.
Montagut, Sánchez-Valle, Castellví, Rami & Molinuevo (2010)42	IV	• n (AD)= 1. AD stage: not mentioned. • Technique and task used: images belonging to five semantic categories. Use of the written word, whenever necessary. • Treatment method: individual. • Frequency and duration of the sessions: four weekly sessions of, approximately, 15 minutes. • Treatment period: 20 sessions. • Work with caregivers involved: no. • Use of associated medication: no.	The participant with AD showed little change in naming ability over time, and a slight increase in vocabulary relative to previous results.
**Face-name association**			
Clare, Wilson, Carter, Roth & Hodges (2002)23	III	• n (AD)= 11. AD stage: mild. • Technique and task used: mnemonic selection, cue vanishing and errorless learning. Use of photographs. • Treatment method: individual. • Frequency and duration of the sessions: not mentioned. • Treatment period: six sessions. • Work with caregivers involved: no. • Use of associated medication: yes.	Significant improvement in naming. The gains were maintained throughout the first six months. Decline after 12 months; however, scores were superior to baseline.
Bier, Linden, Gagnon, Desrosiers, Adam, Louveaux et al. (2008)18	III	• n (AD)= 15. AD stage: mild. • Technique and task used: spaced retrieval, errorless learning and cue vanishing (target methods), trial and error with explicit memory and implicit memory instructions (control methods). List of images and names. • Treatment method: individual. • Frequency and duration of the sessions: two weekly session of 45 minutes each. • Treatment period: five weeks. • Work with caregivers involved: no. • Use of associated medication: no.	The five techniques used were effective for the face-name association in the AD group.
Clare, Wilson, Carter & Hodges (2003)24	IV	• n (AD)= 1. AD stage: mild. • Technique and task used: mnemonic assistance, spaced retrieval and errorless learning. Use of photographs. • Treatment method: individual. • Frequency and duration of the sessions: for the practice at home, once a day. • Treatment period: six months. • Work with caregivers involved: no • Use of associated medication: no.	Significant improvement in face-name association and the gains were broadly maintained over time. The follow-up indicated maintenance (superior scores in relation to baseline).
**Memory cards during conversation**			
Mcpherson, Furniss, Sdogati, Cesaroni, Tartaglini, et al. (2001)27	IV	• n (AD)= 2. AD stage: severe. • Technique and task used: mnemonic assistance. • Treatment method: conversation in pairs. • Frequency and duration of the sessions: 10 minutes of conversation. • Treatment period: 6 weeks. • Work with caregivers involved: no • Use of associated medication: no	One participant with AD showed an increase in the time spent within the topic of conversation.
Bourgeois (1993)28	IV	• n (AD)= 6. AD stage: moderate to severe. • Technique and task used: mnemonic assistance. • Treatment method: conversation in pairs. • Frequency and duration of the sessions: five minutes of conversation, three times a week. • Treatment period: not mentioned. • Work with caregivers involved: no • Use of associated medication: not mentioned. Educational level: not mentioned	There was an improvement in the number of statements within the topic, a reduction in ambiguity and in the production of unfruitful sentences. Non-generalized results.
**Communicative training of caregivers of people with AD**			
Bourgeois, Burgio, Schulz, Beach & Palmer (1997)44	IV	• n (AD)= 7. AD stage: not mentioned • Technique and task used: caregiver training. • Treatment method: individual. • Frequency and duration of the sessions: three cycles of intervention, (each cycle) four weeks of pre-intervention, 12 weeks of intervention and 24 weeks of reevaluation. Three hours of individual workshop. • Treatment period: 16 weeks a year. • Work with caregivers involved: yes. • Use of associated medication: not mentioned.	Reduction of repetitive verbal behavior and maintenance during follow up. The control group had an increase in repetitive verbal behavior rates.
**Conversational interaction**			
Chapman, Weiner, Rackley, Hynan & Zientz (2004)17	II	• n (AD)= 54. AD stage: mild to moderate. • Technique and task used: intervention groups combined with medication and medication only. • Treatment method: group. • Frequency and duration of the sessions: weekly of one and a half hours. • Treatment period: eight weeks. • Work with caregivers involved: no. • Use of associated medication: yes.	Non-significant scores for discourse relevance (cognitive intervention and medication intervention groups). Decline in the medication intervention group.
**Instrumental communicative activity of daily living**			
Perilli, Lancioni, Singh. O'Reilly, Sigafoos, Cassano et al. (2012)43	IV	• n (AD)= 4. AD stage: moderate. • Technique and task used: computer aid. • Treatment method: individual. • Frequency and duration of the sessions: once or twice a day. • Treatment period: 50 sessions. • Work with caregivers involved: no. • Use of associated medication: yes.	Increase in the average of independent phone calls made. The average dialogue time per session varied across the participants from five to six minutes.

EL: evidence level; AD: Alzheimer's disease.

Different cognitive rehabilitation techniques were used. Among the most common methods, the following were observed: errorless learning,^11^^,^ 18 ^-^ 25 clue vanishing,^18^^,^ 20 ^,^ 23 space retrieval,^18^^,^ 24 ^,^ 26 trial and error^18^^,^ 22 and the use of reminiscence (mnemonic) assistance.^19^^,^ 23 ^,^ 24 ^,^ 27 ^,^ 28 Five of the twelve studies that used these cognitive techniques aimed at intervention for different cognitive skills (including language), three stimulated face-name association, two used lexical-semantic therapy and two made use of memory cards during conversation.

We observed that 68.96% of the manuscripts reported the stage of AD included in their samples. Among these studies, 50% included participants at the mild stage of cognitive decline in AD, 36.84% mild-to-moderate stage, 5% moderate, 5% moderate-to-severe, and 5% included participants at the severe stage of cognitive decline in AD. Notably, only one intervention study, which used memory cards during conversation, included participants at the advanced stage of AD. A total of 65.51% of the study samples conducted interventions with individuals who had over 10 years of education. Only one study included individuals with a lower educational level of 2 to 6 years of education.^29^ Possible beneficial effects were reported in both of these studies which included samples with AD at advanced stage and participants with lower educational levels.

Most studies conducted interventions with a frequency of two weekly sessions, and each session lasted, on average, for 60 minutes with sessions given during a period of one to four months. Five studies were classified as evidence level (EL) I (17.24%), three as EL II (10.34%), nine as EL III (31.03%),and twelve as EL IV (41.37%). Most of the papers found were classified as having medium-to-low scientific evidence (Chart 1).

Regarding results obtained, we observed that the interventions conducted, in the case of most studies (79.31%), showed benefits and significant results for at least one communicative skill. Among the positive results observed were increase and/or maintenance effects for scores on language and communication evaluations. Some 20.68% confirmed the maintenance of positive results through follow-ups after intervention; however, among these, only 16.66% compared participants against a control group without intervention. Among the main skills that showed an improvement on post-intervention scores were naming, fluency, and conversational skills. Of the results that showed maintenance of skills, oral and written understanding prevailed. Among the studied interventions, 20.68% showed no effects, that is, the performance of participants declined after intervention. Considering the four studies classified as EL I, three reported improvements and one showed prevailing decline (Chart 1).

Types of interventions and results according to classification were as follows.

### Intervention involving several cognitive skills (including language).

These intervention studies involved the stimulation of many cognitive skills in addition to language, such as time-space orientation, attention, memory, executive function, visuospatial and problem resolution-related skills. Out of the nine studies included, one was classified as EL I,^29^ one as EL II,^26^ one as EL III,^30^ and six as EL IV.^11^^,^ 19 ^,^ 20 ^,^ 25 ^,^ 31 ^,^ 32 The results of these studies showed improvement in the post-intervention performance of caregiver-patient interaction skills (EL I),^29^ verbal fluency (EL I, II, III and IV),^25^^,^ 26 ^,^ 29 ^,^ 30 face-name association (EL II),^26^ semantic categorization (EL III),^30^ phonological discrimination (EL III),^30^ oral understanding (EL III and IV),^20^^,^ 30 ^,^ 32 reading (EL IV),^11^^,^ 32 writing (EL IV),^11^^,^ 25 naming (EL IV)^11^ and repetition (EL IV).^20^ Information regarding the maintenance of skills was reported in studies with EL IV for language,^19^^,^ 25 articulation, automatic speech and written naming^19^^,^ 31 scores. In relation to the maintenance of positive effects during follow-ups after intervention, three studies (EL IV)^20^^,^ 25 ^,^ 32 conducted this analysis. One indicated the maintenance of language results;^25^ two showed a decline in the effects after 12 months.^20^^,^ 32 Post-intervention improvement prevailed in these studies, providing evidence of the efficacy of this type of intervention.

### Language activities integrated with physical activities.

The studies included in this category focused on using language tasks associated with physical training. Language activities conducted ranged from conversation^33^^,^ 34 to the use of different language stimulation exercises^35^^-^ 37 and responses to autobiographical questions.^37^ The physical activities executed included walking,^33^^,^ 34 stretching and aerobic exercises,^37^ as well as weight resistance exercises.^35^^,^ 36 Out of the five studies included in this category, two were classified as EL I^33^^,^ 34 and four as EL III.^35^^-^ 38 Three studies showed post-intervention communicative improvements (one classified as ELI and the other two as EL III). The improvement shown in the study with EL I was related to social and global communicative skills.^33^ In the other two studies (EL III), the improved abilities were discourse skills.^35^^,^ 36 Post-intervention maintenance of discourse, naming and verbal fluency skills was observed in studies classified as EL III.^35^^,^ 36 There was no evidence of maintenance of positive effects after follow-ups of this type of intervention, since none of the studies conducted a follow-up. It is noteworthy that the positive results were not generalized to all the participants of the samples investigated in these studies. Despite the presence of improvements and the maintenance of discourse skills in the studies referred to above, two of the studies^36^^,^ 37 showed decline in communication after intervention. In these studies (one classified as EL I and the other as EL III), post intervention performance revealed a reduction in the number of words and information units expressed, as well as a reduction in conversational concision and increased use of vague nouns. In addition, testing of linguistic skills showed deterioration in naming.^36^^,^ 37

### Lexical-semantic therapy.

Studies that were classified into this category used activities such as tasks which requested the patient to detect semantic relationships between items, name figures, interpret the meaning of isolated words, as well as words within sentences and within stories. From the six studies included in this category, two were classified as EL I,^39^^,^ 40 one as EL II,^41^ two as EL III^21^^,^ 22 and one as EL IV.^42^ With regard to results, four studies^21^^,^ 22 ^,^ 39 ^,^ 41 showed post-intervention improvement in naming skills (EL I, II and III)^21^^,^ 22 ^,^ 39 ^,^ 41 and phonemic, verbal and semantic fluency (EL I).^39^^,^ 40 Only one study (classified as EL IV)^42^ showed no post-intervention effects, with few and insignificant changes from baseline for naming skills and vocabulary.^42^ Only two studies investigated the maintenance of positive effects in follow-ups after intervention. One of these^24^ showed that participants maintained the positive effects, whereas the other^39^ showed decline over time. The positive outcomes mentioned were obtained in studies with EL I, II and III, providing important evidence suggesting the efficiency of this type of intervention.

### Face-name association intervention.

Three studies were included in this category, and the purpose of this type of intervention was to learn names of people. Names that were difficult for the patient to memorize and that belonged to people who were relatively close to them represented in photographs were selected in these studies. Two of the studies were classified as EL III^18^^,^ 23 and one as EL IV.^24^ Although the ELs are low, the three studies showed positive results, with significant improvement in face-name association skills. In relation to the maintenance of positive effects on follow-ups after the interventions, two studies^23^^,^ 24 involved follow-ups, indicating maintenance of the results. Some post-intervention decline was also detected in scores; however, scores were greater than at pre-intervention baseline.

### Instrumental communicative activity of daily living.

one study explored progressive decline in instrumental activities of daily living, and focused on training communication in an ecological context.^43^ This study proposed an intervention in which visual and hearing inputs were used as mnemonic support for phone calls. The results indicated an increase in the average of phone calls made independently, given that at baseline none of the participants made calls independently. Although positive results were found, EL was classified as low (IV). No evidence was shown regarding the maintenance of the results with a follow-up after the end of the intervention.

### Communicative training of caregivers.

One study conducted this type of indirect intervention, training caregivers of people with AD to use communicative and mnemonic strategies in conversations.^44^ The strategies were based mainly on the use of signals and sentences that were syntactically simple, in order to reduce the repetitive verbal behavior of people with AD. The caregivers were previously trained and instructed to deploy the reduction program whenever there were repetitive verbalizations. The results were positive with reductions in repetitive verbal behavior after intervention. The study showed evidence of maintenance of the results in a follow-up after the end of the intervention; however, the EL of this investigation was classified as IV.

### Intervention based exclusively on conversational interaction.

One study,^17^ classified as EL II, used exclusively discourse interaction activities among speakers with AD. In order to increase relevant verbal content in the discourse of participants with AD, the intervention focused on conversations about personal events. The results indicated a non-significant improvement in discourse relevance. Although the improvement was only slight, the control group with no intervention showed a significant decline in discourse production. Similarly, the result of the follow-up conducted post-intervention indicated maintenance of discourse scores for the intervention sample. Although mnemonic aids were not used in this study, results obtained with memory cards described in the study below indicate that perhaps discourse within this kind of conversation context may show significant improvement.

### Use of memory cards during conversation.

Memory cards are materials containing photographs and short sentences that may be used as a mnemonic aid during communication between the person with AD and his or her communicative partner. Two studies conducted an intervention based on the use of memory cards.^27^^,^ 28 Positive effects were obtained in these studies, such as increased time spent speaking on the topic, reduction in ambiguity, improvement in discourse and social content, increase in the number of statements and shifts, and a reduction in repetitions. However, the two studies that investigated this technique were classified as having low EL (IV). In addition, although positive results were obtained, the findings of both studies could not be generalized to all participants of the sample. Follow-up measurements were also not conducted in order to verify the maintenance of effects post-intervention.

## DISCUSSION

This systematic review revealed many different types of intervention that may have a beneficial impact on the communicative skills of people with AD. Most of these studies had positive effects. However, studies with high evidence levels are only being produced on a small scale, and studies with medium and low scientific evidence still prevail. The most common methodological limitations included: small samples, lack of randomization of the participants in the allocation of samples and absence of comparison with a control group.

The studies that showed greater levels of evidence were the lexical-semantic interventions and those with language integrated with physical activities. However, considering these two types of intervention, we concluded that the results of the lexical-semantic approach showed greater reliability of efficacy. This conclusion was due the fact that studies that investigated the effect of language intervention integrated into physical activities did not always show improvements. In fact, one of these studies showed decline in discourse skills after four months of three weekly sessions of this modality.

The intervention involving several cognitive skills (including language) showed predominantly beneficial results, including studies with high evidence levels. Although most of these studies obtained language improvement effects, a considerable amount obtained only maintenance effects while those studies that conducted follow-up showed decline after a period of time. This type of intervention needs to be more accurately investigated, since low-evidence studies of this kind of therapy still prevail.

There is a lack of investigation of certain types of intervention that yielded positive results: face-name association interventions, approaches that use memory cards during conversation, communicative training of caregivers and training of communicative instrumental activities. The small number of studies is concomitant with the use of low evidence level methods to investigate the effects of these approaches. A larger number of studies that explore these interventions and the use of methodology that can assure higher evidence levels may offer better parameters to attest whether or not these approaches are effective.

Similarly, interventions based exclusively on conversational interaction need further investigation. Although the evidence level of the study that used this approach was relatively high (II), the results in terms of therapeutic efficacy were not significant. It is possible that the short duration and the low intensity of the conversational intervention of this study were not sufficient to produce improvement. Further studies should be conducted to investigate the effect of conversational interventions, since this type of task is ecological and can better capture the effects of therapy in daily life. However, it is known that the progress of research on the effects of discourse interventions faces challenges, since discourse tasks differ significantly, as does the type of analyses done. The use of standardized tasks should help increase evidence for this promising approach.

In relation to the maintenance of positive results of language interventions over time, it is fundamental that further research verify the maintenance of post-intervention effects by conducting follow-ups. Considering those studies which conducted follow-ups, the studies that found maintenance only slightly outnumbered those that found decline. However, it is not possible to draw solid conclusions about the duration of intervention effects due to the small number of studies that conducted follow-ups.

There is growing demand for attesting therapeutic efficiency to validate the use of clinical practices related to interventions to maintain or rehabilitate cognitive and communicative skills in dementia. It is important to highlight that conducting research on the effects of interventions on the communication of dementia populations is a major challenge. However, professionals working in this area should consider that the development and acknowledgement of communicative interventions will increase considerably with the adoption of carefully designed methods, which should include blind evaluators, investigate groups with a significant number of participants, and include a cognitively paired control sample. However, since this area of intervention is still in development, even studies with low evidence levels offer important contributions by paving the way for new approaches that should then be investigated more robustly and rigorously.

Additionally, the use of standardized tasks and measures of discourse will allow higher data accuracy and generalization of ecological approaches. Since it may be harder to capture improvements with discourse analysis, it is fundamental to establish clearly defined discourse markers. The importance of developing evaluation protocols that may encompass the complexity of communicative changes in AD is clear. In order for the effect of therapies to be demonstrated, it is important that therapists have efficient instruments designed to measure changes that are highly related to daily communication.

Global aspects of communication and quality of life measures related to the functionality and pleasure of communicating should also be considered in intervention studies. There is evidence that, even in advanced stages of AD, there is a great emotional need for communication.^45^ During the entire progression of the disease, it is necessary for communication to be a part of life for people with AD. Thus, therapeutic approaches should also take into consideration the emotional well-being of subjects. We observed a major gap in the literature in relation to the investigation of interventions with patients at advanced stages of AD. In this respect, it is noteworthy that therapeutic programs should focus not only on improving cognitive and linguistic skills related to communication. Intervention studies should also look toward possible changes in the well-being of patients at advanced stages of AD and their family members and caregivers. Additionally, research should invest more in investigating the efficacy of orienting family and caregivers on the use of verbal and non-verbal communication strategies and on the adaptation of environments.
